# The Field Automatic Insect Recognition‐Device—A Non‐Lethal Semi‐Automatic Malaise Trap for Insect Biodiversity Monitoring: Proof of Concept

**DOI:** 10.1002/ece3.70642

**Published:** 2024-11-28

**Authors:** Juan A. Chiavassa, Martin Kraft, Patrick Noack, Simon Walther, Ameli Kirse, Christoph Scherber

**Affiliations:** ^1^ Weihenstephan‐Triesdorf University of Applied Sciences Merkendorf Germany; ^2^ Thünen Institute of Agricultural Technology Braunschweig Germany; ^3^ Weihenstephan‐Triesdorf University of Applied Sciences Freising Germany; ^4^ Leibniz Institute for the Analysis of Biodiversity Change (LIB), Museum Koenig Centre for Biodiversity Monitoring and Conservation Science Bonn Germany; ^5^ Bonn Institute for Organismic Biology University of Bonn Bonn Germany

**Keywords:** biodiversity monitoring, e‐traps, iNaturalist, insects, sensor‐based monitoring, smart and automatic traps

## Abstract

Field monitoring plays a crucial role in understanding insect dynamics within ecosystems. It facilitates pest distribution assessment, control measure evaluation, and prediction of pest outbreaks. Additionally, it provides important information on bioindicators with which the state of biodiversity and ecological integrity in specific habitats and ecosystems can be accurately assessed. However, traditional monitoring systems face various difficulties, leading to a limited temporal and spatial resolution of the obtained information. Despite recent advancements in automatic insect monitoring traps, also called e‐traps, most of these systems focus exclusively on studying agricultural pests, rendering them unsuitable for monitoring diverse insect populations. To address this issue, we introduce the Field Automatic Insect Recognition (FAIR)‐Device, a novel nonlethal field tool that relies on semi‐automatic image capture and species identification using artificial intelligence via the iNaturalist platform. Our objective was to develop an automatic, cost‐effective, and nonspecific monitoring solution capable of providing high‐resolution data for assessing insect diversity. During a 26‐day proof‐of‐concept evaluation, the FAIR‐Device recorded 24.8 GB of video, identifying 431 individuals from 9 orders, 50 families, and 69 genera. While improvements are possible, our device demonstrated its potential as a cost‐effective, nonlethal tool for monitoring insect biodiversity. Looking ahead, we envision new monitoring systems such as e‐traps as valuable tools for real‐time insect monitoring, offering unprecedented insights for ecological research and agricultural practices.

## Graphical abstract

The FAIR‐Device, a novel nonlethal field tool, combines semi‐automatic image capture and AI‐based species identification to monitor insect biodiversity. In a 26‐day proof‐of‐concept evaluation, it recorded 431 individuals across diverse taxa, demonstrating potential as a cost‐effective solution for high‐resolution insect monitoring.

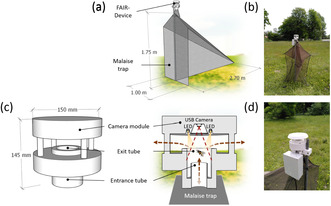

## 1 Introduction

A large number of recent studies have documented or at least suggested potentially severe declines in biodiversity at various spatiotemporal scales, particularly in insect taxa such as flying insects, wild bee pollinators, and butterflies (Hallmann et al. 2017; Ceballos, Ehrlich, and Dirzo 2017). At the same time, we lack consistent and efficient approaches to assess large‐scale spatial patterns in insect population trends (Montgomery et al. 2020). However, many current large‐scale monitoring efforts, like the Global Malaise Trap Program (Centre for Biodiversity Genomics 2024), aim to develop and standardize protocols for the acquisition and analysis of detailed temporal and spatial information on terrestrial arthropod communities. Malaise traps are broad‐taxa spectrum traps (Uhler et al. 2022) capable of catching hundreds of insect specimens per day (Skvarla et al. 2021; Montgomery et al. 2021), which may be problematic from a species conservation perspective. Additionally, the sheer volume of specimens makes it practically impossible to manually sort the masses of insects collected by such traps. For example, after 15 years of the Swedish Malaise Trap Project, only about 1% of the traps caught have been identified by experts (Karlsson et al. 2020). Although new techniques such as metabarcoding can significantly improve and reduce the time of sample processing (Piper et al. 2019; Iwaszkiewicz‐Eggebrecht et al. 2023), this method is not without its challenges and limitations. Metabarcoding requires specialized equipment and trained personnel for DNA extraction and sequencing (Montgomery et al. 2021) and faces issues such as methodological biases due to the lack of standardized protocols (Iwaszkiewicz‐Eggebrecht et al. 2023; Sickel et al. 2023). Furthermore, difficulties in accurately determining species abundances (Sickel et al. 2023) and the scarcity of comprehensive reference databases (Montgomery et al. 2021; Iwaszkiewicz‐Eggebrecht et al. 2023) present additional hurdles. Therefore, it is highly probable that the development of real‐time monitoring systems and nonlethal traps will be a priority for many future biodiversity monitoring endeavors around the world.

In recent years, advancements in sensors, processors, network infrastructure, and information management systems—such as artificial intelligence (AI) and big data—have paved the way for a multitude of new monitoring devices that provide unprecedented access to environmental information (Preti, Verheggen, and Angeli 2021; Porter et al. 2009; Li et al. 2019; Cardim Ferreira Lima et al. 2020). Among these innovations, electronic traps (e‐traps) have emerged as a standout solution. Designed to replace traditional insect monitoring methods, these traps employ various technologies to gather data on insect populations, behavior, and diversity. These traps provide real‐time, wirelessly transmitted data that undergo continuous analysis. Leveraging advanced sensor technologies, e‐traps are at the forefront of monitoring solutions, particularly those that include image acquisition and processing (Preti, Verheggen, and Angeli 2021; Sciarretta and Calabrese 2019; Ascolese et al. 2022; Høye et al. 2021).

AI technologies play a crucial role in this context, with recent breakthroughs in deep learning, computer vision, and image processing achieving remarkable accuracy. As a result, manual in situ observations and routine laboratory sample processing can now be surpassed (Sciarretta and Calabrese 2019; Høye et al. 2021). However, most e‐traps primarily focus on studying agricultural pests, tailoring their trap and bait designs to specific species or a limited range of insects (Rydhmer et al. 2022). This specialization makes them less suitable for monitoring highly heterogeneous insect communities and poses challenges for conservation science due to their often lethal nature. In summary, while technological progress has revolutionized insect monitoring, there remains a critical need for nonlethal, nonspecific solutions capable of comprehensively assessing insect diversity across ecosystems.

Recently developed noninvasive systems that utilize cameras and AI algorithms for insect detection and identification provide an exception to lethal automated monitoring devices. For instance, the Insect Detect DIY camera trap is designed to video monitor a colored surface through tracking and recognition algorithms (Sittinger et al. 2023; Sittinger 2024). This surface is optimized to attract mainly hoverflies (*Syrphidae*) and Hymenoptera. The trap is based on cost‐effective off‐the‐shelf hardware components combined with open‐source software. Light traps that use computer vision have also been presented (Bjerge et al. 2021; Yao et al. 2020). The Automated Moth Trap (AMT), for example, uses a combination of light and a camera to attract and detect nocturnal moths. The video‐captured moths are then identified, tracked, counted, and classified using image processing algorithms. Both the Insect Detect and AMT trapping devices are important steps toward building nonlethal insect monitoring devices; however, automated monitoring systems for flying insects in general have been less frequently available so far.

The use of smart mobile devices as biodiversity monitoring tools has also gained popularity in recent times, thanks to the development of smartphone apps for taxonomic identification. These apps often offer AI image identification functionalities for species classification, and many are constantly improving as algorithms and computing power advance (van Horn et al. 2021). Two of the most popular taxonomic identification applications are iNaturalist (https://www.inaturalist.org) and ObsIdentify (https://observation.org), with millions of downloads on Google Play (Apps on Google Play, 2023a, 2023b) and Apple's App Store (App Store 2023a, 2023b). Both apps allow users to record, identify, and share observations of wild organisms with the platform's community. The community actively curates observations by adding identifications to those made by others, enhancing data accuracy. These platforms also provide a way for users to connect, build their knowledge, and contribute to science.

Biodiversity assessments using mobile apps are usually considered noninvasive and expand the possibilities of conservation monitoring in near‐real time, especially compared to lethal insect traps. However, the main purpose of such portals is the opportunistic recording of species occurrences rather than structured ecological monitoring (Høye et al. 2021). This community science data, based on photography‐collection approaches such as those collected on the iNaturalist platform, has been shown to be less reliable and more biased than collection‐based monitoring (Turley et al. 2024). These biases can stem from several factors, including the uneven geographic distribution of observations, with more data coming from urban and accessible areas compared to remote regions. Additionally, photographers tend to capture more visually appealing or larger species, leading to an underrepresentation of smaller or less conspicuous taxa.

Here, we introduce the FAIR‐Device, a broad‐taxa and nonlethal field stationary device based on semi‐automatic image capture and species identification by artificial vision through the iNaturalist platform. Our objective was to develop a low‐effort and cost‐effective monitoring system that allows identifying and counting insects in the field noninvasively and at high temporal resolution.

Although many e‐traps have been presented in recent years to automatically count and/or identify insects, we here aim at a truly nonspecific monitoring device that directly builds upon the traditional Malaise trapping systems.

Specifically, our study has the following aims:

1. To analyze whether our development of an insect monitoring system allows for real‐time and nonlethal assessment of a wide range of taxa of different species.
2. To evaluate the taxonomic classification through the processing of acquired images using the iNaturalist AI system.
3. To conduct an exploratory analysis combining insect monitoring data with other datasets, such as climatic variables, to demonstrate its potential for gaining new insights into insect population dynamics.

We analyze the performance of the device in terms of the quality of the captured data. The behavior of insects and their exit patterns are analyzed. Additionally, since future plans include capturing flapping frequency (also called wing beat frequency, WBF) data, we evaluate how often individuals fly or at least flap their wings inside the device. Second, we evaluate the efficiency of the iNaturalist AI system for taxonomic classification. Since only a proof of concept was performed, no simultaneous monitoring with conventional traps was performed for comparison. However, the level of identification of the system is analyzed for each observation. In addition, the community's rectifications or ratifications and the accuracy of their reviews are also evaluated. Finally, in the third approach, we conduct a preliminary analysis to explore the potential of real‐time insect data combined with climatic variables. An in‐depth analysis awaits future research. Overall, we demonstrate the potential of combining field‐based broad‐taxa nonlethal monitoring devices with an AI‐based curated taxonomic image classification platform to allow for automatic and real‐time monitoring of insects, applicable also to future larger‐scale monitoring programs, especially in cases where lethal traps are not an option.

## 2 Materials and Methods

The FAIR‐Device was primarily designed to replace the collection bottle attached to the outlet of a Malaise trap. Malaise traps are versatile passive net‐traps capable of catching a broad spectrum of insect taxa, with a preference for flying insects, mainly Diptera and Hymenoptera (Skvarla et al. 2021). The nets, made of variable thickness depending on the target species, are arranged in an open tent‐like form to intercept passing flying insects. Intercepted insects tend to climb or fly upward to escape (Malaise 1937), and they are funneled to the peak of the trap and eventually find their way into a collector jar with a lethal and preservative liquid. To allow nonlethal trapping, the design of the FAIR‐Device's casing allows insects to exit the device. Additionally, a computer surveillance system monitors changes in the field of view of the camera inside the device. However, unlike Malaise traps, which also capture microinsects for analysis, our device, given its design and technical limitations, focuses on insects larger than 3 mm. When the camera detects the movement of any entering insect above the size limit of 3 mm, a video recording will be generated until the movement stops. The generated video is subsequently saved to the SD card (Secure Digital card) of the device. The housing design, electronic components, and the method used to count and identify insect species are described in the following sections.

### 2.1 Trap System

**FIGURE 1:**
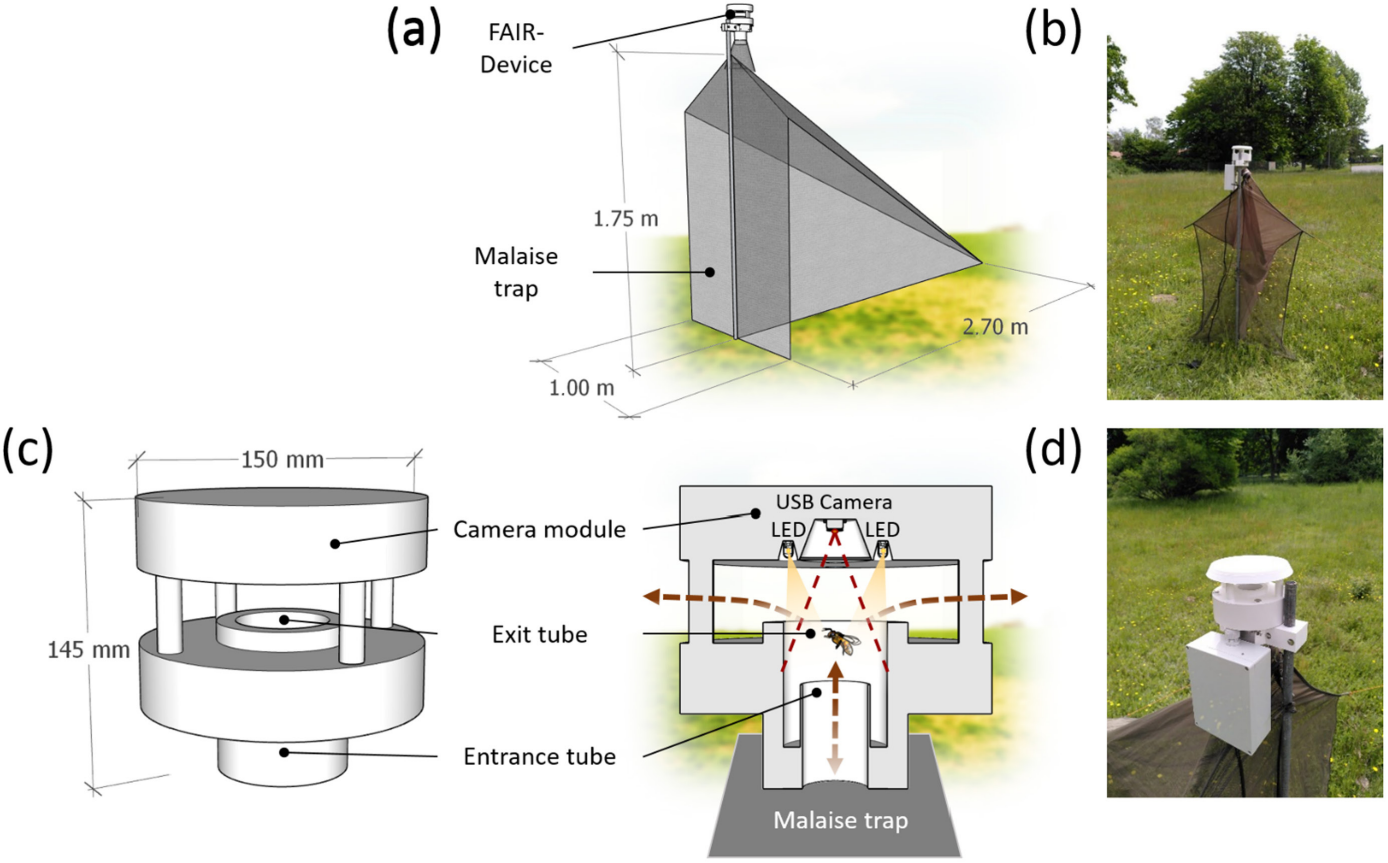
Design of the FAIR‐Device. (a) Schematic view of the Malaise trap with FAIR‐Device attached on top; (b) FAIR‐Device in the field, Summer 2021, Thünen Institute Braunschweig; (c) Close‐up of the FAIR‐Device with camera module, exit hole, exit, and entrance tubes; (d) Close‐up of the device in the field.

The trapping system tested to bring insects into the FAIR‐Device was a Malaise trap developed by the dipterologist M. Bartak (Czech Agricultural University, Prague) (Bartak 2023) with changes made by Bioform (Bioform, Nürnberg, Germany) (Bioform 2023) (Figure 1). The trap had dimensions of 270 cm in length, 100 cm in width, and 175 cm in height. The net material consists of UV‐resistant 1‐mm double‐thread fabric.

### 2.2 Device Design and Operation Principles

Once intercepted by the Malaise trap and funneled upwards, the insects enter the FAIR‐Device through a 30‐mm wide entrance tube, either by flying or crawling. After reaching the rim of the inlet tube, the insects find themselves in a tube with a larger diameter (50 mm), illuminated from above by four LEDs that provide light to allow image capture and, at the same time, to attract and induce them to move upwards, toward the device's exit hole. Once outside, sunlight reduces the attraction effect of the LEDs. The focus of the camera and the LEDs are directed downwards, against the expected direction of insect movement, leaving this configuration with only a few blind spots. The camera lens was calibrated to focus at the exact distance where the rim of the inlet tube is located (74 mm). The movements generated inside the device automatically activate the video recording. A more detailed exploded view of the device will be described in Figure 1.

### 2.3 Device Components and Auxiliary Systems

The housing components of the device are designed to be fully 3D printed and easy to assemble, which significantly reduces manufacturing costs and allows for dynamic design improvement. The 3D models are available online in the Supporting Information. The components were 3D‐printed with white acrylonitrile styrene acrylate (ASA) filament, a material that is highly resistant to weathering and UV radiation, making it ideal for outdoor applications.

The camera includes a 1.0‐megapixel USB camera (ELP720p camera with 45° M7 objective, model USB100W07M‐MHV45; Ailipu Technology Co. Ltd. Shenzhen, Guangdong, China) connected to a Single Board Computer (SBC). Four 5 mm LEDs (334‐15/T2C3‐2TVC, Everlight Electronics Co. Ltd. NewTaipéi, Taiwan) were used to illuminate the device's interior.

The SBC used was a BeagleBone Black (BeagleBoard.org Foundation, Michigan, USA), a low‐cost, SBC with a 1 GHz ARM Cortex‐A8 processor, 512 MB of DDR3 RAM, and 4GB of eMMC flash storage. This community‐supported development platform can run Linux and other operating systems and is commonly used for prototyping and experimentation in fields such as robotics, automation, and the Internet of Things (IoT) (BeagleBoard.org Foundation 2022). The SBC's storage was a 64 GB SD card (SanDisk Ultra, Western Digital UK Ltd., Guildford, Surrey, UK).

The support systems of the prototype were designed to be as simple and reliable as possible, as our aim at this proof‐of‐concept stage was not to achieve fully autonomous operation in field conditions. The power supply was connected to the conventional power grid (230 V) and converted to low‐voltage direct current (5 V) to power the SBC. The SBC was connected to the computer via a medium‐distance WLAN network (EAP110‐Outdoor access point, TP‐Link Corporation Ltd., Tsim Sha Tsui, Hong Kong).

To obtain real‐time weather data, an autonomous weather station was installed approximately 6 m away. The WS501‐UMB Smart Weather Sensor (G. Lufft Mess‐ und Regeltechnik GmbH, Fellbach, Germany) recorded data on temperature, relative humidity, air pressure, wind direction, wind speed, solar radiation, and precipitation (using the Lufft rain gauge WTB100) every 15 min.

The cost of the device, excluding the Malaise trap, is estimated at approximately 210 Euros.

### 2.4 Automatic Image Capture and Manual Processing

**FIGURE 2:**
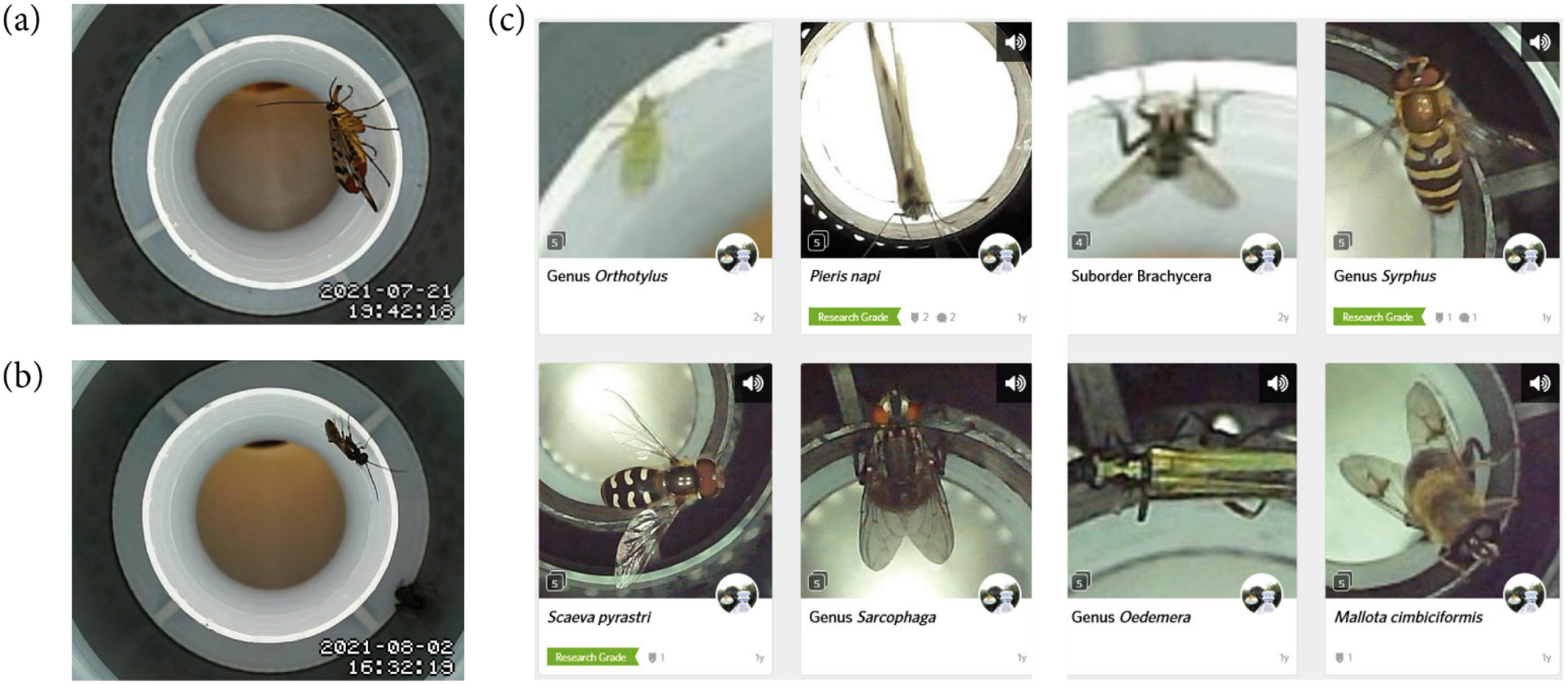
Raw images and image database. (a) A scorpionfly (*Panorpa communis* L., Mecoptera: Panorpidae) photographed by the camera module on 21 July 2021; (b) an unidentified parasitoid wasp (Hymenoptera: Ichneumonoidea), 2 August 2021; (c) Video frames uploaded to inaturalist.org (screenshot taken 7 July 2023), including suggested taxonomic identifications. Note the “research grade” badges, indicating high certainty in taxonomic identification.

The camera module was operated using a Linux distribution program called MotionEye (Crisan 2022) that was installed on the BeagleBone. The app is written in Python and is a web frontend for the motion daemon, a program mainly developed to manage surveillance cameras. Captured images can be viewed live‐streamed and can also be saved as photos or videos (Figure 2). It also has widely configurable functionalities for both motion detection to trigger image capture and region of interest masking. MotionEye was configured to record videos instead of images (.mp4 format) since in this way the behavior of the insects inside the device could be observed, as well as knowing with greater precision and less ambiguity the moment of entry and exit of individuals from the device. To minimize the use of the capacity of the SD memory unit, a video resolution of 352 × 288 pixels was used, considered the lowest possible that still allows obtaining acceptable morphological details of the recorded insects. As it is planned to implement an AI tracking system in the future, it was decided to set the camera to the highest supported recording speed, that is, 30 fps.

**FIGURE 3:**
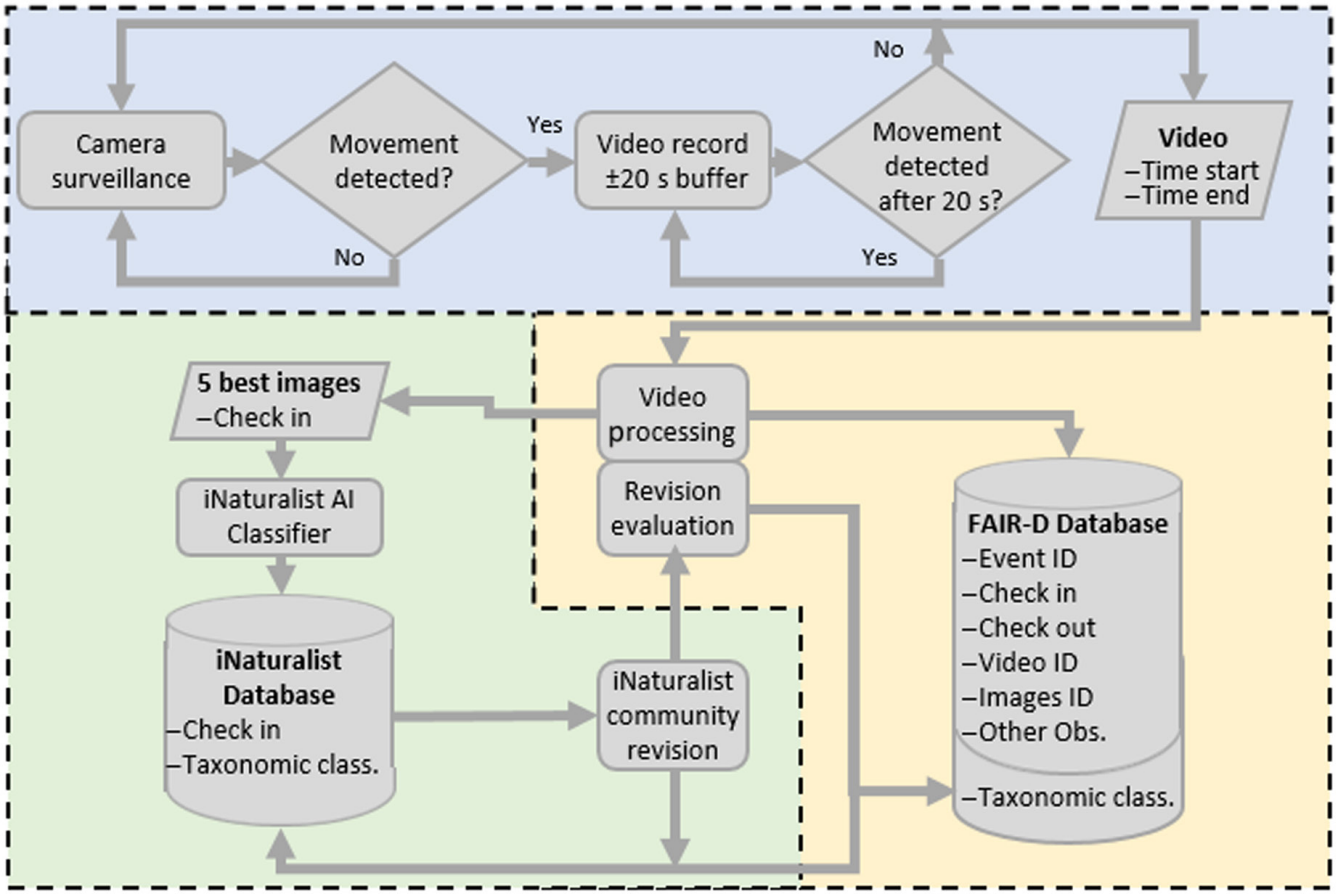
Flowchart of the management of data captured by the device. The blue surface corresponds to the operation of the device in the field, the yellow one to the manual process, and the green one to the process on the iNaturalist platform.

The processing pipeline for the automatic image capture and subsequent database creation for this study is detailed in the flowchart shown in Figure 3. The camera's monitoring system was programmed to detect movement when there were changes in the image of at least 5% and at most 50% of the pixels. This last parameter was set up to avoid, for example, that changes in light (most pixel changes) generate false positives of movement. Likewise, it was established that 120 frames be recorded before and after the movement event (buffer) to determine the exact moment of entry and exit of the individual. If after 20 s no new movement was detected, the recording ended, and the video was saved to the SD card with an event start timestamp. Since the test was carried out on the field without constant supervision, we did not evaluate the incidence of false negatives, that is, the entry of an insect into the device without it being detected.

The videos automatically saved by the FAIR‐Device were analyzed manually. The first step consisted of eliminating the false positives, that is, videos where movement was erroneously detected in the absence of an insect. Next, each video containing insects was visually interpreted. Even though two or more individuals may have appeared in the same video, each appearance was treated as a single event. The interpretation process involved annotating relevant information to feed an event database and capturing at least 5 of the best‐quality image frames of each individual for subsequent taxonomic classification. Image quality was defined based on images and whether the images were (i) not blurred due to the insect's movement and (ii) overall sharpness. The information of each event registered in the database consisted of the ID of the event, the ID of the video(s) with the same individual, the precise time to the second of its entry (check‐in) and exit (check‐out), and the ID of the directory to which the extracted images had been saved. Likewise, other observations arising from the video analysis were recorded, such as the insect's exit direction (again through the entrance or toward the exit) and whether the insect flapped its wings or took flight.

### 2.5 Taxonomic Classification of Insects

The taxonomic classification of the insects recorded on video was carried out with the support of the AI identification system of the iNaturalist platform. For image data, iNaturalist uses computer vision systems trained on other users' photos and identifications to provide automated taxonomic ID suggestions (iNaturalist 2022) that facilitate the identification of the species captured in the observation.

To identify insects using iNaturalist, we uploaded the manually extracted image frames from each individual to the platform. Although automated uploads are not allowed due to a legal requirement on the platform, iNaturalist has developed an application programming interface (API) called VisionAPI that provides direct access to the Computer Vision Model. It allows for automatic image uploads and returns species suggestions based on the iNaturalist computer vision system. However, this feature is not publicly available, and only a small number of select individuals/organizations have fee‐based access for research or use in other citizen science apps.

Based on the quality of the images and the type of species, the AI system identified individuals at different taxonomic levels, displaying one main most probable taxonomic suggestion and eight alternative suggestions. We simulated a completely automatic identification system by taking the main suggestion marked by the AI, even if it did not coincide with our own expert entomological knowledge. If the iNaturalist's AI indicated that it did not have enough confidence in its suggestions, we classified the observation at the lowest taxonomic level that encompassed all the AI's taxonomic suggestions. For example, if the system suggested different species belonging to the order Diptera and Hymenoptera but did not provide a main suggestion, we manually assigned the insect to the class Insecta. Observations that could not be determined as an insect were discarded.

While we used the iNaturalist AI system for all classifications, we published (submitted) about a third of them (154 out of 431 observations) in our platform project profile to open the possibility for analysis by the community. This approach of submitting only a portion of the classifications aimed to avoid excessive repetition of detected insect species while still ensuring a balanced representation. We included common and unique taxa (one‐time detected), choosing to submit those we believed would best illustrate the diversity observed during our study. Although our upload criteria did not follow a rigorous methodological framework, we believe the fraction submitted to iNaturalist is a representative sample of the total observations. In iNaturalist, each submitted classification is available to the platform community for ratification, rectification, or refinement at the most accurate possible taxonomic level. The data quality assessment in iNaturalist evaluates the accuracy of observations, which start as “casual” and progress to “needs ID” if they have a date, georeferenced coordinates, photos, or sounds and are not of organisms captive or cultivated/bred by humans. Observations achieve “research grade” when over two‐thirds of the community agrees on a species‐level ID or lower unless the community votes that the Community Taxon can no longer be improved, in which case a subfamily‐level ID can also achieve “research grade.” Observations revert to “casual” if these conditions are not met or if the community deems the location inaccurate or the evidence insufficient (iNaturalist Help 2024a). *Research Grade* observations are considered high‐quality and are shared in a scientific database that is free to use for research purposes (Boone and Basille 2019).

In the community reviews of our observations, we evaluated system accuracy by calculating the number of agreed‐upon or disagreed with classifications (IDs). We used a similar evaluation system to iNaturalist (iNaturalist Help 2024b). Observations were counted as “disagreed” if the majority (more than 2/3) suggested an ID on a different branch of the taxonomic tree. For example, if we classified a specimen as *Panorpa vulgaris* and the community suggested *Panorpa communis*, this would be marked as a disagreement (annotated as “disagree”). Conversely, if an observation was classified at the genus level (e.g., *Panorpa*) and the community suggested a species‐level ID (e.g., *P. communis*), this correction would be considered an agreement, noting the more specific taxonomic level (annotated as “agree: more specific taxon”). Similarly, if the community suggested an ID at a broader taxonomic level (e.g., Family Panorpidae), it would still be considered an agreement, but with noted inaccuracy (annotated as “agree: more general taxon”). Finally, as a ground truth test to evaluate the overall accuracy of the classifications, we conducted a *post hoc* review of the observations reviewed by the iNaturalist community, agreeing with or disagreeing with the new IDs. These data were taken as the definitive classification in our project database. Data regarding this analysis can be found online in the Supporting Information.

Identifications were recorded in our database using class, order, suborder, superfamily, family, subfamily, genus, and species. Other intermediate minor ranks such as tribe, subtribe, subgenus, and subspecies were disregarded.

### 2.6 Study Site

The study was carried out near the city of Braunschweig, located in the east of the state of Niedersachsen (Lower Saxony), Germany. The device was installed in the test field of the Thünen Institute of Agricultural Technology (52°17′27.6″ N 10°26′18.3″ E). Local vegetation is a naturalized grassland of 0.4 ha, surrounded by small broadleaf forests and crop fields.

### 2.7 Field Tests

Field tests were conducted using a single device in July and August 2021 (see Table S1 in the Appendix S1). The housing design, camera module, and software operation were tested over a period of 26 days and 300 h of effective operation. A subperiod of 10 days was selected (between 24th July and 3rd August) over which the device had a continuous operation time. This period is used here to analyze the data in combination with climatic information. During these 10 days, there were *N* = 125 observations with operation hours from 6:00 a.m. to 7 p.m. (CST Time Zone).

### 2.8 Statistical Analyses

Data were analyzed using R 4.2.3 (2023‐03‐15 ucrt ‐‐ “Shortstop Beagle”) operated via RStudio (2023.06.0 Build 421, 2009–2023 Posit Software, PBC). Insect counts were analyzed at a range of temporal scales: To analyze daily abundance changes, abundances were summed for each hour (0–24 h).

Insect abundances vs. date and stay duration inside the trap were analyzed using a generalized additive model (R package mgcv (Wood 2017)) with a negative binomial family with either date or log‐transformed stay duration as an explanatory variable. Log transformation of stay duration was necessary to account for the highly unequal spread of values in x direction (leverage). The smooth term in the generalized additive model was a penalized thin‐plate spline (“ts” in the gam call (Wood 2003)). The optimal degrees of freedom were estimated using generalized cross validation (option “select = T” in the call to gam).

Responses of insect abundances to temperature and wind speed were analyzed using generalized linear models with negative binomial errors, implemented in the glm.nb function in the MASS library in R (Chambers et al. 2002). The explanatory variables were check‐in date and time, air temperature, and wind speed, all modeled using a B‐spline basis of order 4.

Insect order composition over time (dates) was analyzed using multinomial models, implemented in the multinom() function in the nnet package in R (Venables and Ripley 2002). The check‐in times of insects in the trap were used; to run the model, these times were transformed to numeric and later back‐transformed to dates again. The explanatory variable was the numeric check‐in time. A B‐spline of order 3 was used to account for nonlinearity.

## 3 Results

### 3.1 Device Design Performance

Throughout the test period, 942 video files (24.8 GB of data) were recorded, out of which 431 insect individuals were manually registered, with about 16 individuals per day. Despite relatively low resolutions to avoid overloading the SBC, the resulting images were clear enough to distinguish the shapes, textures, and colors of the insects (Figure 2a). By configuring the camera to record at its maximum possible speed (30 fps), the videos had a sufficient number of frames to produce sharp images in most cases, even when capturing insects in constant motion.

The MotionEye application also met the specifications of properly detecting the entering insects and, in most cases, fully recording the event (89.7% of the cases). However, some difficulties were encountered with the generation of false positives due to the limitations of the pixel‐change‐based motion detection system. Despite different settings, about 15% of the total recorded videos were false motion detections. They were usually triggered by sudden changes in light, and their occurrence could not be completely prevented.

**FIGURE 4:**
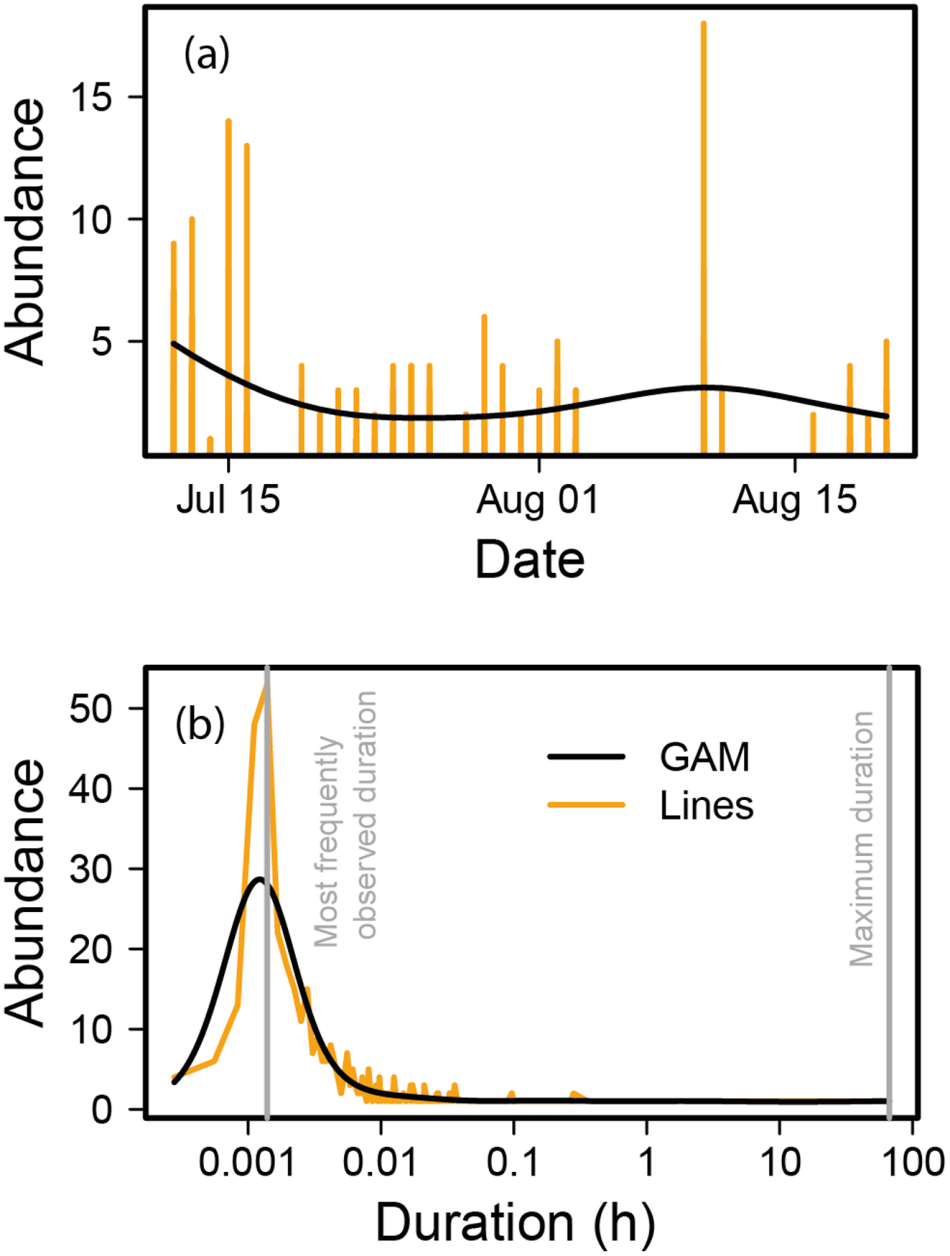
(a) Mean insect abundance per hour per day throughout the operating period of the trap in 2021; (b) average duration of stay of insects inside the traps (in hours). The orange line connects the observations, the smooth black line is a generalized additive model fit. The gray vertical line*s* indicate the most frequently observed *and the maximum* duration.

Over the course of the operation period, insect abundance per hour and day fluctuated around 0–5 individuals (2.6 ± 0.2; Figure 4a). The penalized thin‐plate spline function was of order 3.7 (Chi^2^ = 34.9, *p* < 0.005, *N* = 163).

Although the behavior of each insect varied, they all eventually left the device. However, on some occasions, this took several hours, with the longest stay being 66:49 h (Figure 4b) for an ant queen (*Formica sp*.). The most frequently observed stay duration was 5 s (Figure 4b). The corresponding penalized thin plate spline for abundance against stay duration (in hours) had 7.442 *df* (Chi^2^ = 451.8, *p* < 0.001, *N* = 140). When leaving the device, 82.8% of insects exited as intended, while 6.9% returned to the entrance, and 10.3% of their actions were unrecorded. Additionally, 73% of the insects showed flight activity at least once, usually on their way to the exit. The taxonomic order with the greatest tendency to fly was Diptera with 89% of the cases. There were other orders whose individuals did not fly, even though they had wings, as was the case for Orthoptera, Mecoptera, and Neuroptera.

### 3.2 Insect Taxonomy

On the iNaturalist platform, 154 out of the total classifications were made public (Figure 2c), which is approximately 36%. There were two cases in which the AI system classified moths as bird species, due to the small size of the specimens and the unusual angle at which they were captured by the camera. These observations were discarded and were not counted as part of the detected individuals. Of the total 154 observations made public in iNaturalist, only 93 (about 60%) were reviewed by the community. Of the reviewed observations, approximately 46% reached the *Research Grade* identification level.

The classification performed by the iNaturalist AI was generally agreed upon by the reviewers in 86% of the reviewed observations, which we consider the system's accuracy (see Materials and Methods: Taxonomic classification of insects for the criteria used to consider the IDs suggested by the community as agreed or disagreed). Among these observations where reviewers agreed, a more specific taxonomic level was suggested in approximately 61% of the cases, while a broader taxonomic level was suggested in 10% of the cases. Regarding the *post hoc* review as a ground truth test for evaluating the global accuracy of the classifications, out of 70 revised observations (13 disagreed by suggesting a different ID and 57 considered for taxonomic level change), 64 were validated by us (about 91%). Only 6 reviews (about 9%) were not validated, as we considered the quality of the images insufficient to ratify the suggested classification.

Most of the insects classified were Diptera (63%), followed by Lepidoptera (13.5%) and Hymenoptera (10%). The B‐spline with *df* = 3 for Date in a corresponding multinomial model had Chi^2^ = 90.09, *df* = 24, *p* < 0.001. Within the Diptera order, about 60% were fly species that could be directly related to animal production activities. This abundance is likely to be due to the existing pig and milk production facilities near the study site.

The order of Diptera also had the lowest taxonomic resolution obtained by the iNaturalist AI (Table 1). While around 99% were recognized at the suborder level, only about 56% were categorized at the superfamily level. The Brachycera suborder was also one of the most difficult to identify at lower taxonomic levels by the iNaturalist AI and community.

**TABLE 1:** Identification percentage according to the different taxonomic levels and for the different orders within the study period, determined by iNaturalist's AI identification system and subsequent community review.

Order	Suborder (%)	Super‐family (%)	Family (%)	Subfamily (%)	Genus (%)	Species (%)
Diptera	98.9	55.5	52.2	39.7	28.3	4.4
Hymenoptera	100.0	95.3	81.4	62.8	51.2	11.6
Orthoptera	100.0	100.0	100.0	96.7	96.7	33.3
Coleoptera	100.0	100.0	100.0	100.0	100.0	80.0
Lepidoptera	79.3	79.3	46.6	29.3	27.6	15.5
Mecoptera	100.0	100.0	100.0	100.0	100.0	40.0
Other	87.5	75.0	75.0	75.0	62.5	50.0
**Average**	**96.3**	**68.2**	**60.3**	**48.0**	**39.2**	**12.5**

*Note:* The higher the level of taxonomic precision, the percentage of identification tends to be lower (transition from green, higher, to red, lower).

Because the system identified individuals usually at a different taxonomic level, it is not possible to determine exactly how many different species were identified. Without considering the nonidentifications for the different taxonomic levels, 9 orders, 50 families, and 69 different genera were identified. Tables S2–S4 show all the unique species identified, at the different taxonomic levels and with their respective relative abundance.

**FIGURE 5:**
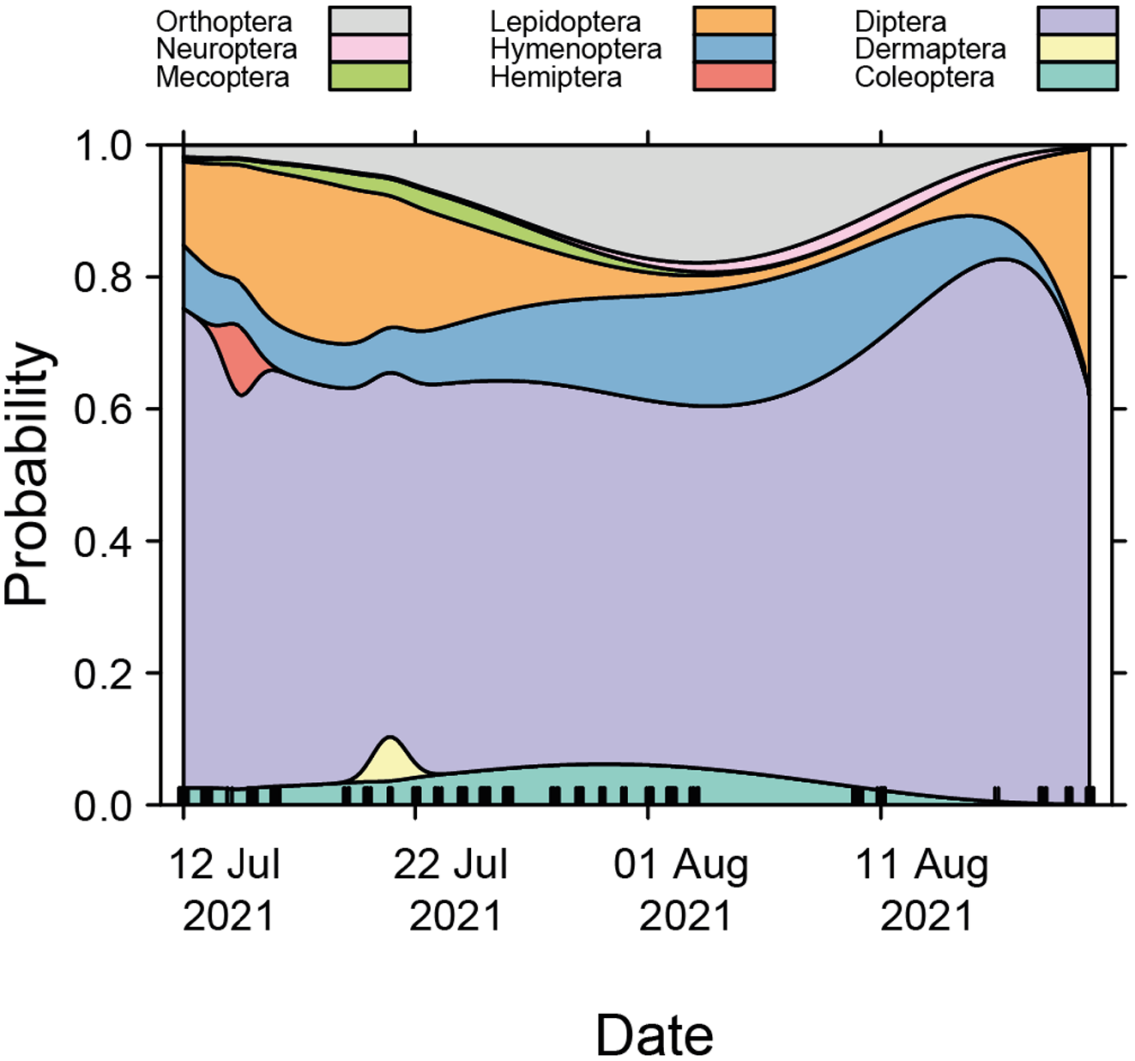
Insect order composition over time. The graph shows predictions from a multinomial model fit using the nnet package in R.

In addition, in an exploratory manner, a multinomial logistic regression model was fitted using insect abundance data to predict the order of insects as a function of time. Figure 5 shows a graphic representation of these probabilities, displaying continuous but fluctuating probabilities for almost all orders. The proportionally larger surfaces reflect the greater probability of appearance for the orders Diptera, Lepidoptera, Hymenoptera, and Orthoptera, in order of magnitude. Hemiptera and Dermaptera, on the other hand, show almost punctual periods with a probability of appearance.

### 3.3 Response to Weather Variables

**FIGURE 6:**
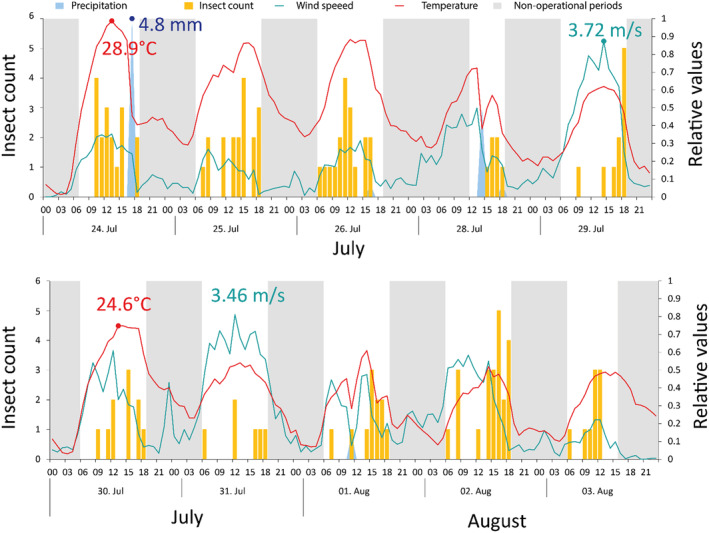
Complex dynamics of insect abundance (yellow), temperature (°C, red), wind speed (m/s, blue‐green), and precipitation (mm, blue), including nonoperational periods of the trap (gray). Note the relative scale to express all values on a common scale. Relative values are scaled to the maximum of each period, as indicated by the exact values given in the upper and lower panels.

**FIGURE 7:**
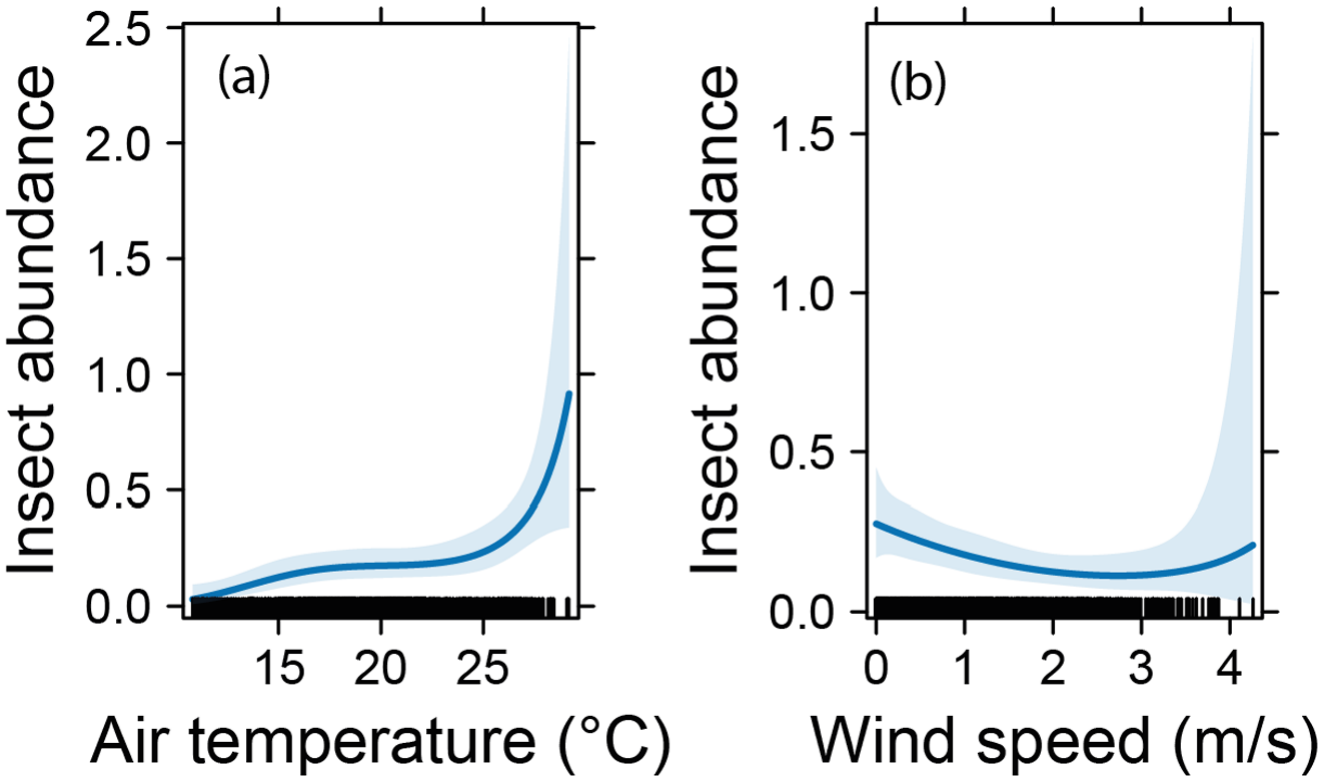
Responses of insect abundance to air temperature (°C) and wind speed (m/s). The lines show back‐transformed predictions from generalized linear models, the shaded areas show the 95% confidence intervals. Rugs on the *x* axes show densities of data points (*N = 3854 time points in total*).

Automated monitoring allows responses of insects to be followed as a function of abiotic covariates such as temperature, rainfall, and wind speed (Figure 6). Generalized linear models with negative binomial errors and B‐spline basis smooths (*df* = 4) for date and time, wind speed, and temperature had highly significant smooth terms for all three explanatory variables (Figure 7a). Insect abundance per 15 min (i.e., the finest possible resolution) increased nonlinearly with air temperature and showed a U‐shaped response to wind speed (Figure 7b).

## 4 Discussion

In the present study, we introduce a novel nonlethal Malaise trap system, revealing a promising avenue for automated insect identification across various taxa and orders. Although the system is not yet fully automated, it achieved an accuracy rate of 86%, which is encouraging. However, approximately 71% of the agreed observations, despite being on the correct taxonomic path, still required some level of correction by the community. This highlights that while the AI system shows potential, significant room for improvement remains. Additionally, to fully assess real taxonomic accuracy, including false negatives, it would have been necessary to catch insects emerging from the device's exit hole and have them identified to species level by taxonomic experts or through DNA barcoding approaches for “ground‐truthing.” These aspects will be addressed in future work.

The FAIR‐Device was only operational between July and August, and its operation times did not always cover the full daylight period. Such interruptions occur in any monitoring system, and certainly, a more continuous and autonomous operation of the device will be aimed for in further developments. Additionally, the device could be supplemented with an intelligent tracking system, where each individual entering could receive a unique ID, avoiding double‐counting or problems with the (rarely occurring) lengthy stays inside the trap. As with Malaise traps in general, they measure activity density, and the radius from which insects are attracted is generally unknown. Further work, for example, with marking and tracking of individuals, would be needed to allow expressing insect observations on a unit area basis (e.g., per square meter).

While the FAIR‐Device captured significantly fewer and overall larger insects compared to traditional Malaise traps, primarily due to motion detector settings and camera resolution limitations, it offers distinct advantages. Our device operates nonlethally and has the potential for full automation features that traditional traps lack. However, we acknowledge that lower monitoring sensitivity, by capturing fewer insects and in fewer different sizes, presents challenges in estimating diversity and pest levels. Specifically, our current approach may overlook smaller insects under 3 mm, which are often abundant and diverse in Malaise trap catches. We therefore believe that it will also be necessary to develop devices capable of capturing information from this spectrum of insects. However, this is challenging, given the technical limitations of video‐based and nonlethal monitoring for such small‐sized species. Complementary approaches, such as using other types of sensors to capture and analyze additional insect biometric information, could address these limitations. For instance, it has been shown that by analyzing the wingbeat frequencies (WBF) of insects, it is possible to identify species with great accuracy (Rydhmer et al. 2022; Potamitis, Rigakis, and Fysarakis 2015; Chen et al. 2014). Solely based on WBF captured through infrared light sensor‐based systems, Potamitis and collaborators were able to detect and recognize small insects, such as fruit flies (Potamitis, Rigakis, and Tatlas 2017) and mosquitoes (Fanioudakis, Geismar, and Potamitis 2018). We believe that these types of systems could be tailored to capture biometric data from insects even under 3 mm, presenting a promising path for future developments.

Additionally, the overall monitoring sensitivity limitations of our development could also be addressed by deploying a network of strategically placed devices, enhancing spatial–temporal resolution in monitoring insect dynamics far beyond the capabilities of current systems. The availability of qualitative and quantitative data on insects at high temporal resolution opens up fascinating new possibilities. For example, in grassland or cropping systems, responses to management events such as grazing, mowing, or pesticide applications could be studied at unprecedented resolution. This is particularly true for designed experiments, where control and treatment plots could be equipped with FAIR‐Devices, allowing simultaneous responses from multiple devices to be measured. The resulting data, some examples of which can be found in this paper (Figures 5, 6, 7), could be used to identify the most effective management strategies for different types of ecosystems, leading to more sustainable and efficient agricultural practices.

Data accumulation poses another significant challenge as we transition to analyzing biodiversity through videos and, to a lesser extent, photos. To address this and to facilitate the complete automation of the device, the development of an efficient AI insect tracking system is crucial. Such a system is currently more essential than creating yet another AI species identification system from scratch. A robust tracking system operating in situ could eliminate the need to send and store large video files. The tracking system would assign a unique ID to each detected individual (thus avoiding duplicate counts), perform a preliminary coarse identification (e.g., at the order level), and extract images from the bounding boxes with the highest identification probabilities. These cropped images, accompanied by metadata (IDs, timestamps, coordinates, etc.), would significantly reduce the file size, requiring less powerful connection networks and much less server storage capacity.

Despite the challenges, we believe the FAIR‐Device's benefits justify its continued exploration and enhancement. These insights and comparisons highlight both the potential and current limitations of our device, paving the way for further improvements. Moving forward, we will focus on both refining the automation of the device and validating the system through comparative testing against traditional monitoring methods.

In terms of taxonomic resolution and coverage, while about 40% of the observations remained unrevised in iNaturalist, the taxonomic identification of individuals that were evaluated by the iNaturalist community was excellent. Given the limited resolution of the images, the resulting classifications are outstanding. Thus, platforms such as iNaturalist and Observation.org with their AI algorithms and community‐based quality control systems are valuable for the initial steps in insect identification from optical monitoring devices. However, the exponential growth in submissions to iNaturalist (Skvarla and Fisher 2023) suggests that the current community‐based approach to curating IDs may not be scalable in the future. If numerous devices operate simultaneously in the field, sending large amounts of data in real‐time, the percentage of user reviews will likely decrease significantly. Additionally, iNaturalist users may become frustrated if automated systems flood the platform with lower quality, potentially repetitive images.

To mitigate these issues, it is advisable to use the iNaturalist AI for initial identifications without submitting the observations, potentially automatically via an API, and have in‐house experts verify these identifications rather than relying solely on the iNaturalist community. Another possibility to avoid a data flood on the platforms is to submit to the community only a limited number of observations generated by the devices. These could be representative samples or cases where human review would be particularly beneficial, for example, when automated classification fails to identify taxa at a useful level. All these strategies could enhance and speed up image processing in automated field monitoring. However, in large projects, where a high flow of data is generated, coordinating with platform administrators will surely be necessary to align with their terms of use and system capabilities.

While the AI algorithms and computer vision capabilities of these platforms are constantly improving, they still struggle with certain identifications, as evidenced by the 75% correction rate by iNaturalist users in this study. It is clear that identification algorithms and computer vision capabilities still need to be improved, especially in cases of low‐quality and low‐resolution images such as those that automated systems can generate, to effectively help mitigate the lack of human supervision. Additionally, future work should focus on developing large image databases for Central European insects taken under field conditions and efficient classification algorithms, which is beyond the scope of the present study.

In a broader scope, we believe it is essential to reconsider the level of information refinement needed based on the proposed objectives, especially given the increasing resources required to reach more refined taxonomic levels. While species‐level identification is commonly used for conservation and pest management, higher‐level taxonomic data as a surrogate for biodiversity (species richness) can still offer valuable insights (Biaggini et al. 2007). Additionally, identifying functional groups from higher taxonomic levels can help estimate ecological roles and interactions within ecosystems (Balzan, Bocci, and Moonen 2014; Traylor et al. 2022). For instance, excluding the problematic order Diptera (in terms of identification accuracies), we achieved more than half of the observations at the genus level (58%) and 74% at the family level. This information remains highly valuable, as in many cases, the family level (or higher) is sufficient to determine the functional group of the identified individual (Lamarre et al. 2016). Therefore, significant inferences could be made about the integrity of an ecosystem for conservation management or the health status of a field for pest management based on automatic monitoring devices delivering data with a taxonomic level sufficient to establish functional groups.

### 4.1 Outlook

Although our device is not yet entirely suitable for field studies, our primary goal was to conceptually evaluate the idea of replacing the Malaise trap's collection bottle with a nonlethal sensor‐based device. We aimed to assess the feasibility of this concept, and based on the results, we believe this objective was largely achieved. However, we acknowledge that technical challenges remain, such as providing an autonomous power source for field operation and ensuring correct data transfer in rural or remote areas. These aspects must be addressed in future developments of the device.

Despite these technical challenges, there are several potential modifications for future versions of the FAIR‐Device. For instance, the device could be mounted on other types of traps, such as vane traps or pitfall traps. Additionally, incorporating a WBF module to record the flying events inside the device could improve identification precision, especially for insects with short stay durations. Furthermore, for night‐time trapping, the device could be converted into a light trap by adding blacklight lamps. Overall, while this first FAIR‐Device version represents only an initial step, we believe it holds transformative opportunities for future biodiversity monitoring.

## 5 Conclusions

We have presented a device with a design and electronics that allows for obtaining quality time‐stamped videos from various species of insects without causing their death. The combination with the iNaturalist platform, thanks to the AI recognition functions and subsequent corrections made by its community, permitted this data to be converted into detailed information about the insect biodiversity of a given site. While there are aspects that still need to be improved or developed, the proposed device has shown promising results for cost‐effective, nonlethal, and high temporal and spatial resolution insect biodiversity monitoring using e‐traps combined with nature identification platforms.

We believe that automated systems capable of monitoring insects in real‐time and 24/7 would open new doors for studying insect populations in ecological research and agricultural production. E‐traps arranged as individual nodes to form networks of devices would enable a highly accurate understanding of the spatial and temporal population dynamics of various insect species. Real‐time information on pest outbreaks, the effectiveness of control or nature conservation measures, and population data on beneficial or endangered insects could be provided by a network of monitoring devices. Local networks could become nodes of larger networks, eventually reaching global coverage (Potamitis, Eliopoulos, and Rigakis 2017). This would allow both agricultural and conservation research communities to benefit from the same data and indicators, leading to better‐informed choices for the long‐term economic and environmental viability of both productive and natural systems.

## Author Contributions

**Juan A. Chiavassa:** conceptualization (equal), data curation (equal), investigation (equal), methodology (equal), software (equal), validation (equal), visualization (equal), writing – original draft (equal), writing – review and editing (equal). **Martin Kraft:** conceptualization (equal), funding acquisition (equal), project administration (equal), writing – review and editing (equal). **Patrick Noack:** supervision (equal), writing – review and editing (equal). **Simon Walther:** supervision (equal), writing – review and editing (equal). **Ameli Kirse:** supervision (equal), writing – review and editing (equal). **Christoph Scherber:** formal analysis (equal), supervision (equal), writing – review and editing (equal).

## Conflicts of Interest

The authors declare no conflicts of interest.

## Supporting information


**Table S1** Operating time (CST) of the FAIR‐Device in July (top) and August (bottom) 2021.
**Table S2** Total unique species identified for Diptera, at the different taxonomic levels and with their respective relative abundance.
**Table S3** Total unique species identified for orders Hymenoptera and Orthoptera, at the different taxonomic levels and with their respective relative abundance.
**Table S4** Total unique species identified for orders Coleoptera, Lepidoptera, Mecoptera, Hemiptera, and Neuroptera, at the different taxonomic levels and with their respective relative abundance.

## Data Availability

The data that supports the findings of this study are available in doi: 10.5281/zenodo.12650557.
