# Supplementary material for: The Field Automatic Insect Recognition‐Device—A Non‐Lethal Semi‐Automatic Malaise Trap for Insect Biodiversity Monitoring: Proof of Concept
Source: Ecol Evol. 2024 Nov 28;14(12):e70642. doi: 10.1002/ece3.70642 (PMC11602669; doi:10.1002/ece3.70642)

### **Appendix**

**Table S1.** Operating time (CST) of the FAIR-Device in July (top) and August (bottom) 2021.

| **Operation** | **Jul 21** | | | | | | | | | | | | | | | | | **Total** |
| --- | --- | --- | --- | --- | --- | --- | --- | --- | --- | --- | --- | --- | --- | --- | --- | --- | --- | --- |
| **Days** | **12** | **13** | **14** | **15** | **16** | **19** | **20** | **21** | **22** | **23** | **24** | **25** | **26** | **28** | **29** | **30** | **31** | **17 days** |
| **Start** | 13:00 | 7:00 | 7:30 | 18:00 | 0:00 | 20:00 | 0:00 | 19:00 | 0:00 | 0:00 | 7:00 | 6:00 | 6:00 | 12:00 | 6:00 | 6:00 | 6:00 | **-** |
| **Finish** | 21:00 | 21:00 | 9:30 | 23:59 | 18:00 | 23:59 | 18:00 | 23:59 | 23:59 | 18:30 | 19:00 | 19:00 | 19:00 | 19:00 | 19:00 | 19:00 | 19:00 | **-** |
| **Effective time** | 8:00 | 14:00 | 2:00 | 5:59 | 18:00 | 3:59 | 18:00 | 4:59 | 23:59 | 18:30 | 12:00 | 13:00 | 13:00 | 7:00 | 13:00 | 13:00 | 13:00 | **201:29** |

| **Operation** | **Aug 21** | | | | | | | | | **Total** |
| --- | --- | --- | --- | --- | --- | --- | --- | --- | --- | --- |
| **Days** | **1** | **2** | **3** | **10** | **11** | **16** | **18** | **19** | **20** | **9 days** |
| **Start** | 6:00 | 6:00 | 6:00 | 15:30 | 0:00 | 12:00 | 18:00 | 15:30 | 0:00 |  |
| **Finish** | 19:00 | 19:00 | 16:00 | 23:59 | 15:00 | 13:00 | 23:59 | 23:59 | 23:59 |  |
| **Effective time** | 13:00 | 13:00 | 10:00 | 8:29 | 15:00 | 1:00 | 5:59 | 8:29 | 23:59 | **98:59** |

**Table S2.** Total unique species identified for Diptera, at the different taxonomic levels and with their respective relative abundance. The abbreviation #N/C stands for “not classified”.


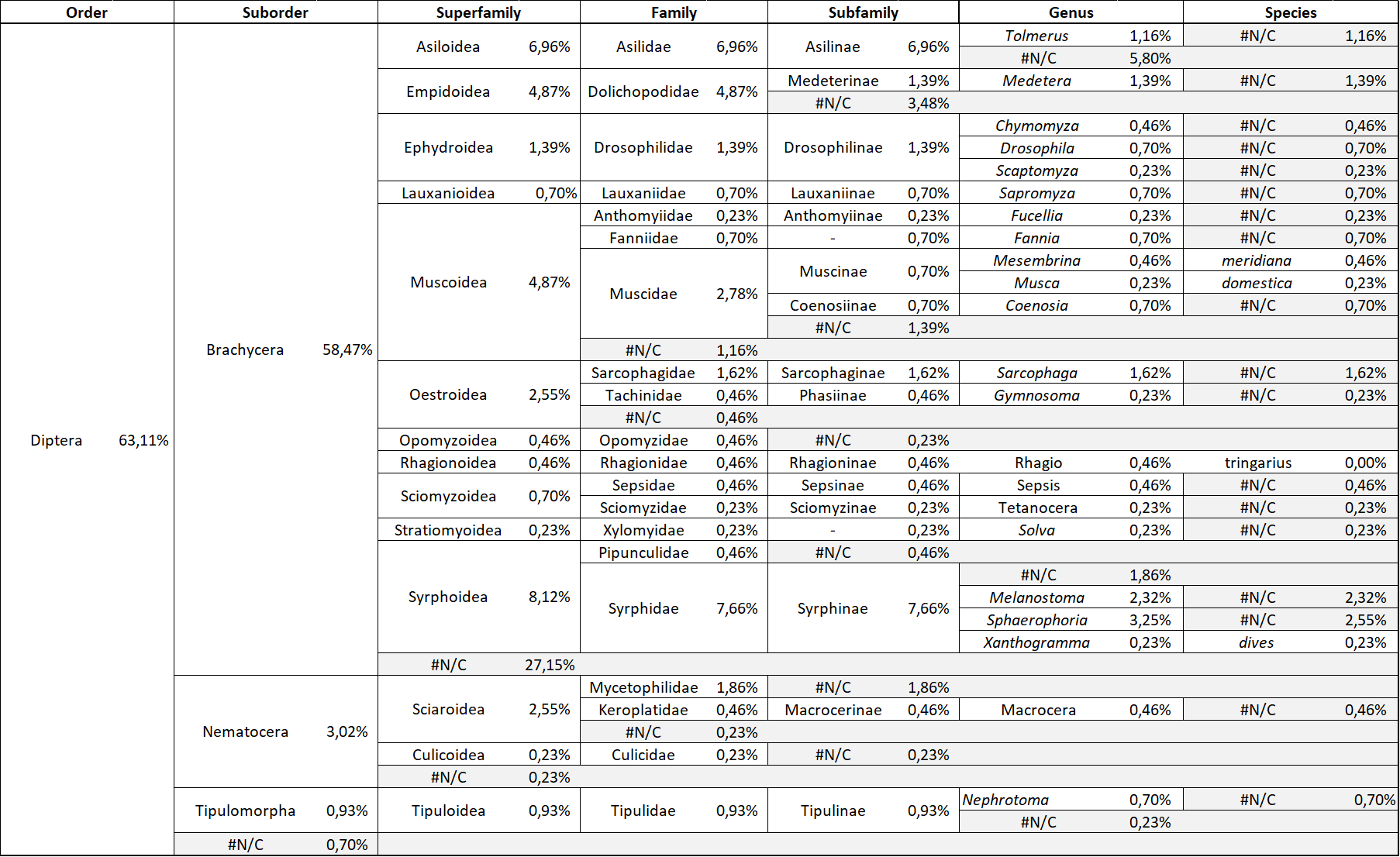


**Table S3.** Total unique species identified for orders Hymenoptera and Orthoptera, at the different taxonomic levels and with their respective relative abundance. The abbreviation #N/C stands for “not classified”.


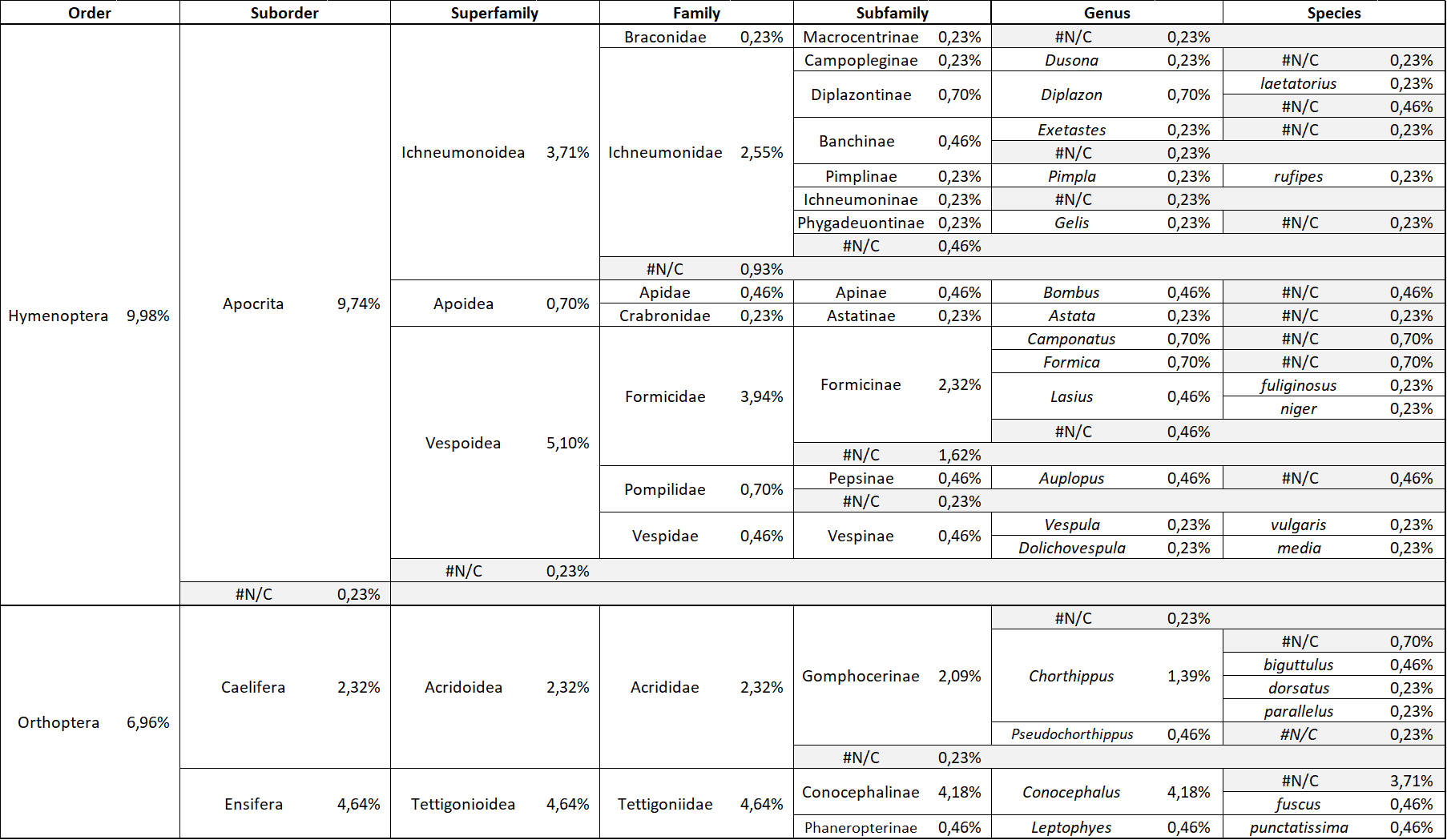


**Table S4.** Total unique species identified for orders Coleoptera, Lepidoptera, Mecoptera, Hemiptera, and Neuroptera, at the different taxonomic levels and with their respective relative abundance. The abbreviation #N/C stands for “not classified”.


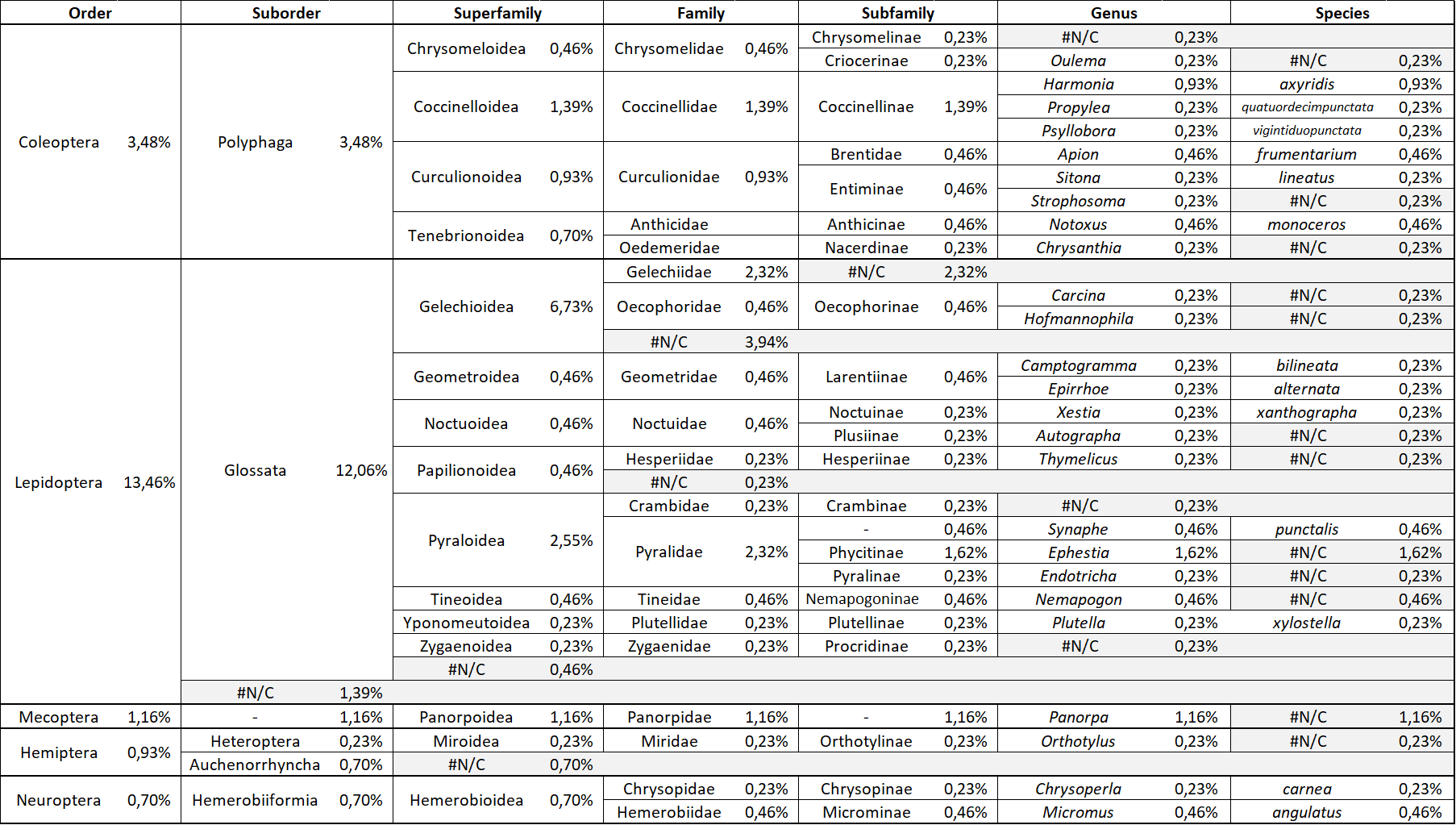

Supplement: Supplementary file 1 — Table S1 Operating time (CST) of the FAIR‐Device in July (top) and August (bottom) 2021. Table S2 Total unique species identified for Diptera, at the different taxonomic levels and with their respective relative abundance. Table S3 Total unique species identified for orders Hymenoptera and Orthoptera, at the different taxonomic levels and with their respective relative abundance. Table S4 Total unique species identified for orders Coleoptera, Lepidoptera, Mecoptera, Hemiptera, and Neuroptera, at the different taxonomic levels and with their respective relative abundance. [file ECE3-14-e70642-s001.docx]
